# Development and application of a nomogram model for predicting the risk of central precocious puberty in obese girls

**DOI:** 10.3389/fped.2024.1421775

**Published:** 2024-08-29

**Authors:** Ren-Hao Huang, Li Yang, Yu Yang, Qing-Bo Xu, Li-Ling Xie, Lan-Fang Cao

**Affiliations:** ^1^ Department of Endocrinology, Jiangxi Provincial Children’s Hosptial/Jiangxi Provincial Clinical Research Center for Children’s Genetic Metabolic Diseases, Nanchang, China; ^2^ Jiangxi Medical College, Nanchang University, Nanchang, China

**Keywords:** central precocious puberty, girls, nomogram, obesity, predictive modeling

## Abstract

**Objective:**

The purpose of this study is to develop and assess a nomogram risk prediction model for central precocious puberty (CPP) in obese girls.

**Methods:**

We selected 154 cases of obese girls and 765 cases of non-obese girls with precocious puberty (PP) who underwent the gonadotropin-releasing hormone stimulation test at the Jiangxi Provincial Children's Hospital. Univariate analysis and multivariate analysis were conducted to identify predictors of progression to CPP in girls with PP. A predictive model was developed and its predictive ability was preliminarily evaluated. The nomogram was used to represent the risk prediction model for CPP in girls with obesity. The model was validated internally using the Bootstrap method, and its efficacy was assessed using calibration curves and clinical decision analysis curves.

**Results:**

In obese girls with PP, basal luteinizing hormone (LH) and follicular stimulating hormone (FSH) levels, as well as uterine volume, were identified as independent risk factors for progression to CPP. In non-obese girls, the basal LH level, bone age, and uterine volume were identified as independent risk factors for progression to CPP. With an AUC of 0.896, the risk prediction model for obese girls, was found to be superior to that for non-obese girls, which had an AUC of 0.810. The model displayed strong predictive accuracy. Additionally, a nomogram was used to illustrate the CPP risk prediction model for obese girls. This model performs well in internal validation and is well calibrated, providing a substantial net benefit for clinical use.

**Conclusion:**

A medical nomogram model of CPP risk in obese girls comprised of basal LH value, basal FSH value, and uterine volume, which can be used to identify those at high risk for progression of CPP in obese girls and develop individualized prevention programs.

## 1 Introduction

Central precocious puberty (CPP) is a common pediatric endocrine disorder characterized by the early development of secondary sexual characteristics in girls before the age of 8 and in boys before the age of 9. The onset of CPP is caused by this early activation of the hypothalamic-pituitary-gonadal axis (HPGA) function. The incidence rate is between 1/5,000–1/10,000, and the incidence rate for girls is 5–10 times higher than that for boys (1). CPP accelerates skeletal maturation, which can result in premature epiphyseal closure and influence adult final height. This can negatively impact their physical and mental health and increase their risk of obesity (2). Obesity is one of the most significant health issues of the 21st century (3). In addition, the prevalence of obesity and CPP has increased over the past few decades (4). Changes in body mass index (BMI) may impact the evaluation of clinical characteristics and laboratory results in children with CPP. In clinical practice, nomogram prediction models that incorporate individual disease characteristics are widely used to predict the diagnosis and prognosis of various diseases. These models contribute significantly to the promotion of personalized medicine (5). In addition, these models have demonstrated greater efficacy than other models in assessing a wide range of disease conditions (6).

## 2 Subjects and methods

### 2.1 Research population

From March 2020 to December 2022, 919 cases of girls with precocious puberty (PP) who were treated at the Department of Endocrinology and Genetic Metabolism of Jiangxi Provincial Children's Hospital were selected retrospectively. The gonadotropin-releasing hormone (GnRH) stimulation test was performed on these cases. Among them, there were 154 cases of obese girls and 765 cases of non-obese girls. The inclusion criteria were as follows: (1) the presence of secondary sexual characteristics, with the Tanner stage of breast development at stage B2 or above at the time of consultation; (2) the age of breast development onset was between 4 and 8 years old; (3) based on the BMI cut-off point standard for children aged 2–18 years old in China (7), obese girls had a BMI ≥ the 95th percentile for their age, whereas non-obese girls had a BMI within the 5th and 95th percentile; (4) complete data were accessible for the cases. In addition, we excluded children who had experienced early onset of menstruation and early pubic hair development. In addition, we excluded children with organic diseases affecting the endocrine glands, peripheral precocious puberty (PP), who had received medication that may have impacted the HPGA prior to consultation, and those with incomplete case data.

### 2.2 Data collection

The medical records, laboratory studies, and imaging studies of the children were examined and collected. General data were collected retrospectively, including age of consultation, disease duration, breast Tanner stage, height (cm), weight (kg), and BMI (kg/m^2^). Laboratory data: basal E_2_, basal luteinizing hormone (LH), and follicular stimulating hormone (FSH) values were obtained, basal LH value is 0.2 IU/L as the critical value, and the LH baseline value is converted into a binary variable (8). And the GnRH stimulation test was completed. The children were administered gonadorelin intravenously at a dose of 2.5 μg/kg, with a maximum dose of 100 μg. Blood samples were collected at 30 min and 60 min after the injection and sent to the laboratory. LH and FSH values were measured twice, and the highest values were recorded as the peak for LH and FSH. Imaging data included bone age film, uterine volume, average right and left ovarian volume, and follicle count. The volume size was determined by multiplying the values of the three diameters by the coefficient 0.5233.

### 2.3 Research methods

Based on the general data, laboratory data, and imaging data of the children, obese and non-obese PP girls were further differentiated into CPP and non-CPP groups, following the guidelines outlined in the *Consensus Statement for the Diagnosis and Treatment of Central Precocious Puberty (2015)* (1) CPP and non-CPP were defined based on whether the HPGA axis function was precociously activated. (1) Early appearance of secondary sexual characteristics: Girls develop secondary sexual characteristics before the age of 8. The first manifestation is the appearance of breast nodules in girls. (2) Linear growth acceleration: The annual growth rate is higher than that of normal children. (3) Advanced bone age: Bone age exceeds the actual age by 1 year or more. (4) Gonad enlargement: Pelvic ultrasound shows an increase in the volume of the girl's uterus and ovaries, and multiple follicles with a diameter greater than 4 mm can be seen in the ovaries. (5) HPGA function is activated, and serum gonadotropins and sex hormones reach adolescent levels. Use the first and fifth items as the CPP inclusion criteria. Among the obese girls, there were 81 children with CPP and 73 children without CPP. Among the non-obese girls, there were 535 children with CPP and 230 children without CPP. Based on the analysis of previous risk factors associated with CPP, 3–6 risk factors are independently associated with the of CPP (9). As a result, the risk prediction model developed in this study included no more than six predictors. Based on the standard requirement for sample size in prediction modeling, there must be least 10 positive outcomes for each predictor variable (10). Therefore, to meet the sample size requirement, it was estimated that 60 children with CPP were required for each group of prediction models. The sample size of this study met the basic requirement.

### 2.4 Statistical methods

We used the SPSS 25.0 and R 4.2.1 software for data organization and statistical analysis. Variables were screened stepwise using univariate analysis (*P* < 0.1) and multivariate logistic regression analysis (*P* < 0.05). The logistic regression model, *P *= 1/(1 + e(*β*_0 _+ β_1_X_1 _+ *β*_2_X_2 _+…+ β_m_X_m_)), was used for the analysis. The variables obtained through multivariate analysis were included in the construction of the predictive model. The predictive efficacy of the model was evaluated using the receiver operating characteristic curve (ROC) and the area under the ROC curve (AUC). The nomogram model was constructed using the regplot program package. The model was validated internally using the Bootstrap method. The calibration of the nomogram model was assessed using the Hosmer-Lemeshow test. The clinical benefit of the predictive model was evaluated through clinical decision curve analysis (DCA).

## 3 Results

In terms of BMI, disease duration, and breast staging, univariate analysis revealed no significant differences (*P* > 0.1) between the CPP and non-CPP groups among obese girls. However, as shown in Table 1, there were significant differences (*P* < 0.1) observed between the two groups in terms of age, E_2_, basal LH value, basal FSH value, bone age, uterine volume, mean ovarian volume, and mature follicles. The difference in the comparison of BMI between the CPP and non-CPP groups in non-obese girls was not significant (*P* > 0.1). However, as detailed in Table 2, the difference between the two groups was significant (*P* < 0.1) when comparing disease duration, age, breast stage, E_2_, basal LH value, basal FSH value, age of the bone, uterine volume, mean ovarian volume, and mature follicles. The variables that passed the appeal screening were analyzed using multivariate logistic regression. The progression to CPP was the dependent variable, while the other variables were defined as independent variables. The results revealed that the LH basal value (OR = 7.47, 95% CI 2.01–27.74), FSH basal value (OR = 1.74, 95% CI 1.15–2.63), and uterine volume (OR = 3.15, 95% CI 1.84–5.38) were predictors of progression to CPP in obese girls, as shown in Table 3. LH basal value (OR = 6.92, 95% CI 4.20–11.39), bone age (OR = 1.40, 95% CI 1.24–1.58), and uterine volume (OR = 1.38, 95% CI 1.16–1.65) were identified as predictors of CPP progression in non-obese girls, as shown in Table 4.

**Table 1:** Comparison of clinical data between obese girls in the CPP and non-CPP groups [M (P25, P75)/*n*(%)].

Research index	CPP group (*n* = 81)	Non-CPP group (*n* = 73)	Z/χ^2^	*P* value
BMI (kg/m^2^)	20.41 (19.76, 21.53)	20.75 (19.55, 22.05)	−0.22	0.822
Disease duration (month)	4.00 (2.00, 6.00)	3.00 (1.00, 10.00)	−1.58	0.115
Age (year)	7.83 (7.42, 8.08)	7.50 (6.79, 8.00)	−2.44	0.015
Breast staging (%)				
Stage B2	36 (44.44)	36 (49.32)	0.37	0.545
>Stage B2	45 (55.56)	37 (50.68)		
E_2_ (ng/dl)	16.16 (11.80, 26.57)	11.80 (8.80, 13.08)	−3.99	<0.001
LH basal value (%)				
≤0.2 IU/L	35 (43.20)	69 (94.52)	46.10	<0.001
>0.2 IU/L	46 (56.80)	4 (5.48)		
FSH basal value (IU/L)	3.59 (2.14, 4.86)	1.87 (1.16, 2.49)	−6.30	<0.001
Bone age (year)	10.00 (8.58, 11.00)	9.00 (8.00, 10.00)	−3.16	0.002
Uterine volume (ml)	3.04 (2.01, 4.79)	1.61 (1.33, 2.07)	−6.47	<0.001
Mean ovarian volume (ml)	1.86 (1.41, 2.49)	1.33 (0.98, 1.63)	−4.97	<0.001
Mature follicles (%)				
No	49 (60.49)	55 (75.34)	3.86	0.049
Yes	32 (39.51)	18 (24.66)		

**Table 2:** Comparison of clinical data between non-obese girls in the CPP and non-CPP groups [M (P25, P75)/*n*(%)].

Research index	CPP group (*n* = 535)	Non-CPP group (*n* = 230)	Z/χ^2^	*P* value
BMI (kg/m^2^)	16.12 (15.04, 17.15)	16.15 (15.02, 17.37)	−0.70	0.484
Disease duration (month)	4.00 (2.00, 7.00)	2.00 (1.00, 6.00)	−4.12	<0.001
Age (year)	7.83 (7.25, 8.08)	7.29 (6.50, 7.92)	−6.73	<0.001
Breast staging (%)				
Stage B2	343 (64.11)	166 (72.17)	4.70	0.03
>Stage B2	192 (35.89)	64 (27.83)		
E_2_ (ng/dl)	15.40 (11.80, 25.13)	11.80 (9.07, 19.12)	−4.20	<0.001
LH basal value (%)				
≤0.2 IU/L	252 (47.10)	209 (90.87)	128.66	<0.001
>0.2 IU/L	283 (52.90)	21 (9.13)		
FSH basal value (IU/L)	2.92 (2.03, 4.14)	2.20 (1.57, 2.82)	−7.40	<0.001
Bone age (year)	9.00 (8.00, 10.00)	8.00 (6.50, 9.00)	−9.67	<0.001
Uterine volume (ml)	2.54 (1.82, 3.70)	1.82 (1.28, 2.40)	−8.99	<0.001
Mean ovarian volume (ml)	1.81 (1.32, 2.43)	1.54 (1.13, 2.01)	−4.42	<0.001
Mature follicles (%)				
No	262 (48.97)	135 (58.70)	6.09	0.014
Yes	273 (51.03)	95 (41.30)		

**Table 3:** Multivariate logistic regression analysis of risk factors for CPP in obese girls.

Research index	β	SE	Wald*χ*^2^	*P*	OR	OR 95%CI	
						Lower	Upper
LH basal value	2.01	0.67	9.01	0.003	7.47	2.01	27.74
FSH basal value	0.55	0.21	6.80	0.009	1.74	1.15	2.63
Uterine volume	1.15	0.27	17.61	<0.001	3.15	1.84	5.38
Constant	−4.38	0.83	28.12	<0.05	0.01		

**Table 4:** Multivariate logistic regression analysis of risk factors for CPP in non-obese girls.

Research index	β	SE	Wald*χ*^2^	*P*	OR	OR 95%CI	
						Lower	Upper
LH basal value	1.93	0.26	57.66	<0.001	6.92	4.20	11.39
Bone age	0.34	0.06	30.59	<0.001	1.40	1.24	1.58
Uterine volume	0.33	0.09	13.35	<0.001	1.38	1.16	1.65
Constant	−3.23	0.50	42.68	<0.001	0.04		

Logistic regression equations were developed using the final screened independent variables, and the regression coefficients and constant terms were obtained from the multivariate logistic regression analysis. The regression equation of the CPP risk prediction model for obese girls was P = 1/1 + EXP (−4.38 + 2.01 × LH basal value + 0.55 × FSH basal value + 1.15 × uterine volume); the regression equation of the CPP risk prediction model for non-obese girls was *P* = 1/1 + EXP (−3.23 + 1.93 × LH basal value + 0.34 × bone age + 0.33 × uterine volume). The CPP risk prediction model for obese girls had an AUC of 0.896. The prediction model had a sensitivity of 77.8% and a specificity of 90.40%, which were both higher than those of the CPP risk prediction model for non-obese girls (Table 5).

**Table 5:** Significance of different logistic regression models for CPP prediction.

Group	AUC	95%CI	Youden's index	Sensitivity (%)	Specificity (%)
Obese group	0.896	0.844–0.947	0.68	77.80	90.40
Non-obese group	0.810	0.778–0.841	0.50	71.40	78.30

**Figure 1:**
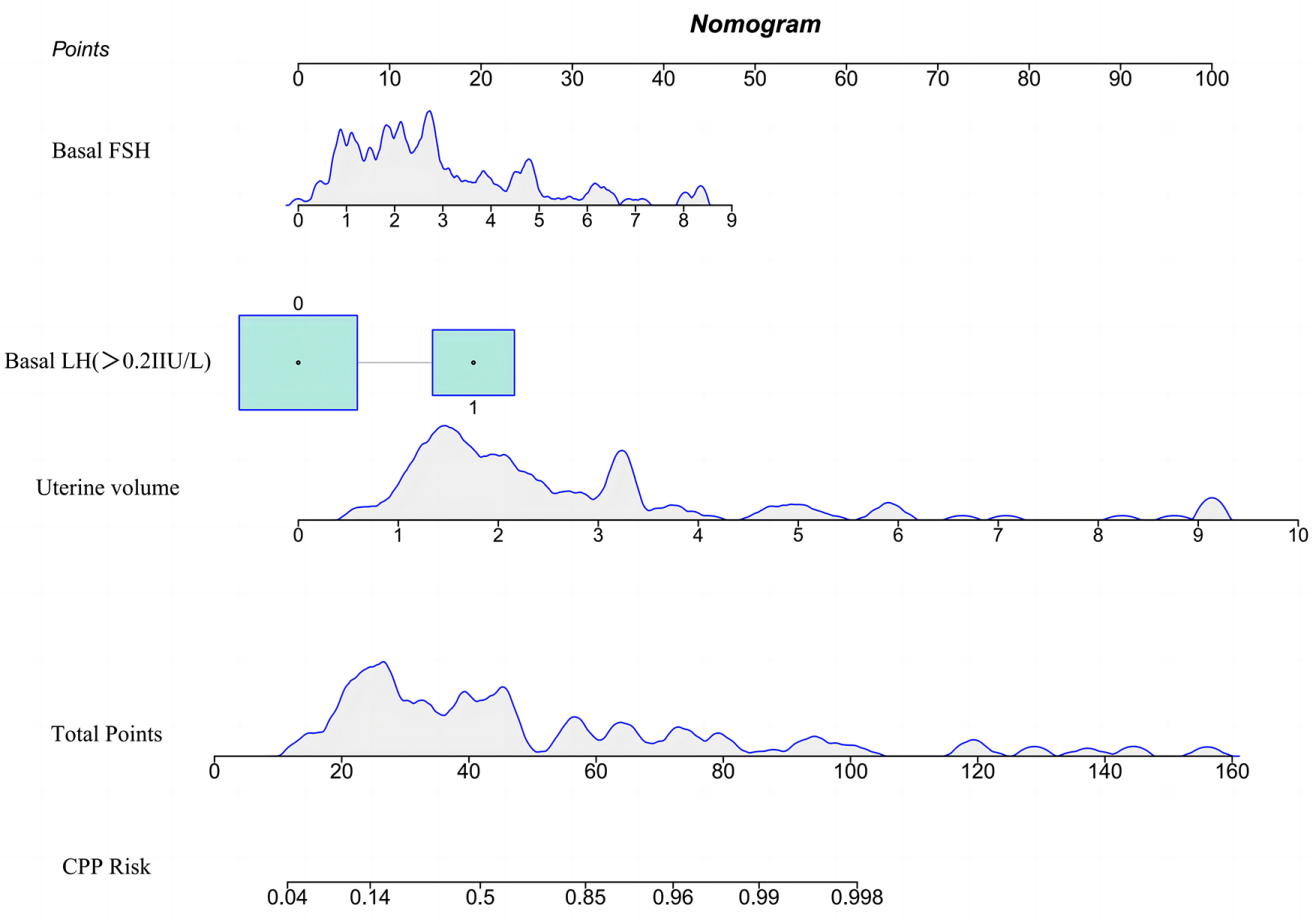
Nomogram of the risk prediction model for central precocious puberty in obese girls.

As depicted in Figure 1, the variables entered in the logistic regression model were used to construct a nomogram model for predicting the risk of CPP in obese girls. For the included variables LH basal value, FSH basal value, and uterine volume, the variance inflation factors were 1.81, 1.84, and 1.28, respectively. These values indicate that there was no multicollinearity among the predictors within the model. During the application of the nomogram model, the total score was calculated by adding the scores on the upper score axis corresponding to each indicator. The predicted probability of the risk of CPP in obese girls was then obtained using the predicted probability on the lower score axis that corresponded to the total score. Assuming that when an obese girl comes for treatment due to breast development, her LH baseline value is less than 0.2 IU/L, FSH baseline value is 2.73, and uterine volume is 1.70 ml, the total score of the patient is 32.9 points, and the risk of progression to CPP is 28.4%, belonging to the low-risk population.

**Figure 2:**
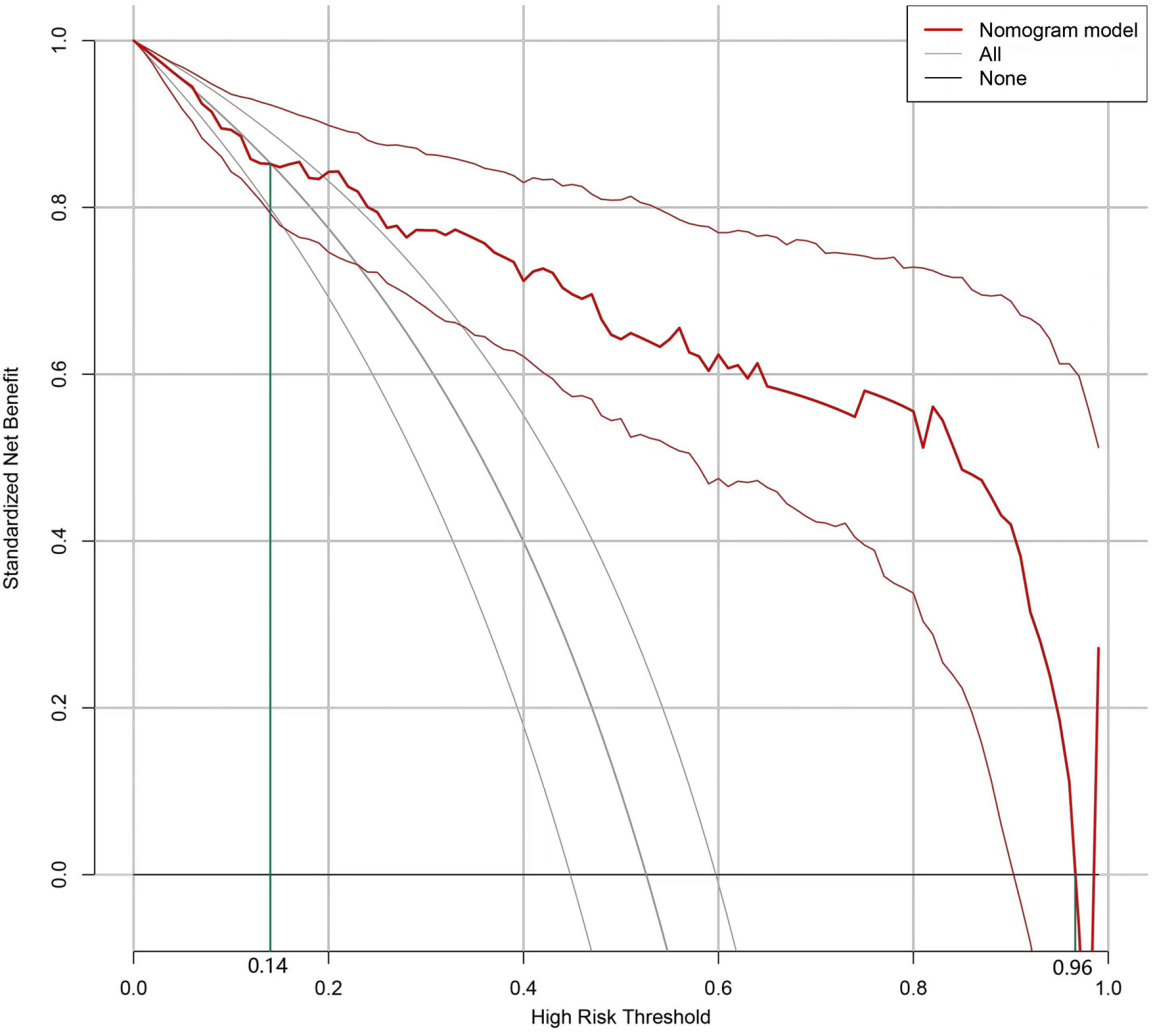
Clinical decision analysis curves of the nomogram prediction model.

The Bootstrap method was used to validate the nomogram model internally. The results revealed that the C-index of the model was 0.895, indicating that the model performed well during the internal validation process. The Hosmer-Lemeshow test results revealed *X*^2 ^= 5.04 (*P *= 0.75), indicating that the predictive model is well calibrated. When the threshold probability of the DCA curve (Figure 2) falls between 14% and 96%, the application of the nomogram risk prediction model established in the current study can provide greater net clinical benefit.

## 4 Discussion

In this study, multivariate logistic regression analysis results revealed that basal LH, basal FSH, and uterine volume were predictors of the occurrence of CPP in obese PP girls. HPGA is routinely measured in the evaluation of children with CPP as its activation correlates with an increase in both gonadotropins—FSH and LH, which are elevated in children with CPP. Basal sex hormone levels serve as important indicators to assist in the diagnosis of CPP, and basal LH levels are significant predictors of positive results in the GnRH stimulation test (11). Due to the deficiency of the GnRH stimulation test, the LH basal value is currently used as an important supplementary index to determine CPP (12). The basal LH value can be used distinguish the stage of pubertal development with reasonable accuracy (13). A study of girls with varying BMIs determined that obese girls had lower LH basal values than non-obese girls (14). However, it was also determined that there was no significant difference in LH basal values between obese and non-obese CPP girls. Moreover, it was hypothesized that progress in bone age (BA) would eliminate the impact of BMI on the determination of basal LH values (15). In addition, other factors such as nutrition, epigenetics, and endocrine disruptors may be associated with the onset of puberty and obesity, and they may affect the interpretation of laboratory results (16). In terms of enhancing the sensitivity of the diagnosis, the predictive efficacy of basal FSH values tends to be weaker and less useful. There are few studies on the association between childhood obesity and FSH. A study conducted by Aydin et al. revealed that an increase in BMI during pubertal development was associated with an increase in prepubertal FSH, and that higher FSH levels increased the risk of obesity and metabolic syndrome (17). The kisspeptin signaling system acts as a crucial link involved in the regulation of gonadotropin secretion during puberty (18). Prepubescent obese girls have higher kisspeptin levels than non-obese girls of the same age, which may be the cause of the factor difference between obese and non-obese girls in the CPP prediction model. As a noninvasive, radiation-free, cost-effective, and reproducible examination that measures various indicators of the uterus and ovaries, pelvic ultrasound has also been widely used in the diagnosis of CPP (19). The results of a study revealed that the pelvic ultrasound indicators of girls with CPP were significantly different than those of normal girls (20). Wang et al. and Yu et al. discovered that uterine volume is the most specific and sensitive ultrasound parameter for diagnosing CPP (21, 22). The uterine volume threshold used to predict CPP, however, fluctuated widely. This variability is associated with the susceptibility of pelvic ultrasound findings to factors such as the skill level of the operator and instrument parameters (23, 24). In addition, the difference in BMI may also be one of the factors influencing the variation in the cut-off value. Combining it with other diagnostic modalities can therefore enhance diagnostic efficacy (25, 26).

The results of this study revealed that the risk prediction model for CPP in obese girls reduced the predictive value of bone age when compared to the non-obese group. Advancement of bone age is one of the most important clinical features in the diagnosis of CPP, and itis a valid predictor for the diagnosis of CPP (27). In patients with CPP, however, bone age and advanced bone age are significantly higher in girls with high BMI and the degree of advanced bone age is positively correlated with BMI (28). Several factors have been found to influence bone maturation. Approximately 25% of children with obesity have advanced bone age. In addition, there is a correlation between advanced bone age and BMI standard deviation scores in girls (29). In univariate analyses, disease duration and breast staging outcomes differed between CPP and non-CPP girls in both the obese and non-obese groups. In the obese group, however, these differences were not statistically significant. In prepubertal and pubertal children, obesity obscures the distinction between bone age and breast stage. This blurring affects the accurate assessment of the disease course and can lead to disturbances when using bone age and breast staging to assess the level of sexual development in obese girls in clinical practice.

In conclusion, the nomogram model developed in this study provides a visual representation of the multivariate results for predicting the risk of CPP in obese girls. This model eliminates the need for complex calculations and enables more intuitive and rapid individual-level predictions. In addition, the predictors in this model are easy to obtain and are all routine examinations for PP girls, which has a high clinical utility and can minimize multiple blood draws for GnRH stimulation tests.

## 5 Conclusion

LH baseline, FSH baseline, and uterine volume are independent risk factors for predicting CPP in obese girls. The predictive performance of the line chart model for predicting the risk of CPP in obese girls constructed in this study is good, at a moderate level, and can be used to identify high-risk populations for the progression of CPP in obese girls and provide personalized prevention plans.

Nonetheless, there are limitations to this study. The relatively small number of participants included in this study may introduce bias into the results, and the retrospective nature of this study in collecting clinical data such as children's dietary habits and lifestyle, may potentially influence the results. Hence, these factors were excluded from the analysis. In addition, this study was conducted at a single center; therefore, it is necessary to validate the results at multiple centers to determine whether they can be generalized to other provinces and regions.

## Data Availability

The original contributions presented in the study are included in the article/Supplementary Material, further inquiries can be directed to the corresponding author.
